# Mucosal-Associated Invariant T Cells Are Depleted and Exhibit Altered Chemokine Receptor Expression and Elevated Granulocyte Macrophage-Colony Stimulating Factor Production During End-Stage Renal Disease

**DOI:** 10.3389/fimmu.2018.01076

**Published:** 2018-05-17

**Authors:** Jennifer A. Juno, Jillian L. M. Waruk, Kathleen M. Wragg, Christine Mesa, Carmen Lopez, Joe Bueti, Stephen J. Kent, T. Blake Ball, Sandra A. Kiazyk

**Affiliations:** ^1^Department of Medical Microbiology, University of Manitoba, Winnipeg, MB, Canada; ^2^Department of Microbiology and Immunology, Peter Doherty Institute, University of Melbourne, Melbourne, VIC, Australia; ^3^National Laboratory for HIV Immunology, HIV/TB Co-Infection Unit, Public Health Agency of Canada, Winnipeg, MB, Canada; ^4^Renal Program, Health Sciences Centre, Winnipeg, MB, Canada; ^5^Melbourne Sexual Health Centre, Department of Infectious Diseases, Alfred Health, Central Clinical School, Monash University, Melbourne, VIC, Australia; ^6^ARC Centre of Excellence in Convergent Bio-Nano Science and Technology, University of Melbourne, Parkville, VIC, Australia

**Keywords:** end-stage renal disease, mucosal associated invariant T cells, latent tuberculosis, inflammation, granulocyte macrophage-colony stimulating factor, CD161

## Abstract

**Background:**

End-stage renal disease (ESRD) is associated with an increased susceptibility to infectious diseases, including infection with *Mycobacterium tuberculosis (Mtb)*. Mucosal-associated invariant T (MAIT) cells recognize vitamin B metabolites produced by many bacterial species, including Mtb, and may play an important role in providing protective immunity against tuberculosis infection in the lung. To date, little is known about MAIT cell frequency, phenotype, or function in ESRD patients.

**Methods:**

MAIT cells, identified by surface marker expression or MR1 tetramer binding, were characterized in 20 ESRD and 20 healthy control participants by multicolor flow cytometry. *Ex vivo* MAIT cell phenotype and cytokine production following PMA/ionomycin, IL-12/IL-18, or *Escherichia coli* stimulation were determined. Monocyte phenotype and plasma C-reactive protein/inflammatory cytokine levels were quantified by flow cytometry, ELISA, and multiplex bead array.

**Results:**

Peripheral blood MAIT cells were significantly depleted among ESRD patients compared to controls by both phenotypic and tetramer analysis and exhibited a loss of CXCR3 expression coupled to increased expression of CCR6 and CXCR6. ESRD was also associated with a shift in MAIT PMA-induced cytokine production away from IFNγ production and toward granulocyte macrophage-colony stimulating factor (GM-CSF) secretion, and a loss of *E. coli*-stimulated tumor necrosis factor α expression. Loss of IFNγ expression was associated with a combination of age, alterations in Tbet and Eomes expression, and inflammatory plasma cytokine levels.

**Conclusion:**

The loss of peripheral blood MAIT cells and associated shifts in tissue homing receptor expression and GM-CSF production may contribute to an immune environment that is permissive to bacterial replication, particularly in the lungs.

## Introduction

Patients with end-stage renal disease (ESRD), the final stage of chronic kidney disease (CKD), exhibit an elevated susceptibility to infectious diseases that has important implications for the clinical care of this population. In particular, patients with renal failure demonstrate high rates of latent tuberculosis infection (LTBI) (1, 2) and are reported to have a 7- to 50-fold increased risk of reactivation of LTBI to active TB disease (ATB) (3–5). Uremia-induced inflammation is thought to be a major contributor to lymphocyte dysfunction and immune impairment in ESRD patients [recently reviewed in Ref. (6)], but most studies of immune function in this population have focused on antigen-presenting cells, B cells, and conventional CD4+ and CD8+ T cells. More recently, non-conventional T cell populations such as invariant NKT (iNKT), γδ, and mucosal-associated invariant T (MAIT) cells have emerged as important bridges between the innate and adaptive immune systems (7). These cells contribute to host immunity against TB infection and a variety of other bacterial and viral pathogens (8, 9), but their function in ESRD patients is poorly described.

Mucosal-associated invariant T cells are typically defined by the expression of the TCR Vα7.2 (TRAV1-2) and CD161 and respond to MR1-restricted vitamin B metabolite antigens produced by some bacteria, including *Mycobacterium tuberculosis* (Mtb) (9, 10) as well as cytokines produced by microbial stimulation such as IL-12 and IL-18. Patients with ATB exhibit depletion of peripheral blood MAIT cells, accumulation of MAITs in the lung, and functional impairment of MAIT cytokine production due to PD-1 expression (11, 12), pointing to the activation and recruitment of these cells to the lung during infection. To date, only a single report has assessed peripheral blood MAIT cell frequency among hemodialysis patients, where cell frequency and absolute count were found to be significantly reduced compared to controls (13). No data are available on whether ESRD is associated with alterations in MAIT activation or phenotype, particularly the expression of chemokine receptors known to be important in tissue homing. MAIT cells typically exhibit high expression of many homing receptors, including CCR5 and CXCR3 (known to be involved in lung homing of T cells) (14–16), and are largely KLRG1+, indicating their differentiated, effector memory status (17). MAIT cells also express a number of cytokines upon activation, including IFNγ, tumor necrosis factor α (TNFα), IL-17 and granulocyte macrophage-colony stimulating factor (GM-CSF), all of which are important in controlling Mtb infection and bacterial replication (18–20). Recently, the expression of certain surface markers, such as CD8 (21), and CD94 (22) were shown to be positively associated with MAIT cell function, but have not been previously characterized in ESRD.

We assessed the frequency, phenotype, and cytokine production profile of MAIT cells from ESRD and non-ESRD controls, either with or without LTBI [defined by the interferon gamma release assay (IGRA)], from a Canadian dialysis cohort. Using multiparameter flow cytometry, we assessed the co-expression of activation and tissue homing receptors on the MAIT population, transcription factor expression, and analyzed cytokine production following PMA/ionomycin, IL-12/IL18, or *Escherichia coli* stimulation. This report confirms the previously published loss of MAIT cells in the peripheral blood of ESRD patients and describes for the first time the altered expression of surface chemokine receptors and increased expression of GM-CSF.

## Materials and Methods

### Setting and Study Participants

The ESRD and healthy control cohorts in this study have been previously described (23, 24). ESRD participants undergoing hemodialysis were recruited from the Health Sciences Centre Renal Program in MB, Canada. Non-ESRD controls were selected from a local TB immunology biobank, which contains cryopreserved PBMC and plasma samples of Manitoban participants with known TB status. All individuals included in the study were HIV, HBV, and HCV uninfected. All participants were administered the Quantiferon-TB Gold In-Tube™ test, and provided informed consent. The study was approved by the Research Ethics Board at the University of Manitoba.

### IGRA Testing

We performed the QuantiFERON-TB Gold In-Tube test™ (Qiagen) according to the manufacturer’s protocol as previously described (23). Briefly, 1 mL of blood was collected into each of three tubes: nil (no antigen), antigen (Mtb peptide antigens ESAT-6, CFP-10, TB7.7), and mitogen (positive control). The tubes were incubated for 16 h at 37°C before being stored at 4°C until processing. Samples were centrifuged at 2,500 × *g* for 15 min, and plasmas were stored at −80°C. IFNγ production in the supernatants was quantified by ELISA. IGRA result was determined by the manufacturer’s recommended cut-off values for positive, negative, and indeterminate responses.

### Peripheral Blood Collection and Processing

Concurrent with the IGRA, peripheral blood samples were collected for plasma collection and PBMC processing. Plasma was frozen in aliquots at −80°C for later cytokine determination. PBMC were isolated by Ficoll gradient separation and cryopreserved for future batch analysis.

### Cell Culture and Stimulation

Cryopreserved PBMC were thawed and rested for 2 h, after which 1 × 10^6^ cells were collected for surface antibody staining as below, and the remaining cells collected for stimulations. PBMCs were stimulated with either PMA (1 ng/mL, Sigma) and ionomycin (200 ng/mL, Sigma) for 16 h, or IL-15 (50 ng/mL) alone, PFA-fixed *E. coli* (MOI of 5) plus IL-15 (50 ng/mL), or IL-12 (50 ng/mL, Peprotech) plus IL-18 (50 ng/mL, RnD systems) for 24 h. GolgiStop and GolgiPlug (BD Biosciences) were added 1 h post-stimulation for overnight stimulations, and 8 h poststimulation for 24 h stimulations. PFA-fixed *E. coli* (DH5α) were prepared according to Ref. (25) by fixing for 5 min in 1% paraformaldehyde and washing.

### Flow Cytometry

For surface phenotypic analysis, thawed cells were collected, washed and incubated with combinations of the following antibodies/dyes for 30 min at 4°C: live/dead aqua or blue (Life Technologies), anti-CD3, CCR6, CD94, CCR2, CD20, CD14, CD16, CD56 (BD Biosciences), CD4, CD8, CD26, CD161, Vα7.2, CXCR6, CCR5, CXCR3, CD69, CD27, CX3CR1 (Biolegend), IL-18Rα (Miltenyi), KLRG1 (eBisociences), and α4β7 [NIH AIDS reagent program (26)]. A full list of antibodies and fluorochromes used are listed in Table S1 in Supplementary Material. Antibodies against the iNKT TCR (6B11, BD biosciences) and pan-gamma delta TCR (Beckman Coulter) were used to exclude those cell populations. Apoptotic cells were excluded by gating on viability dye negative cells. Staining with the human MR1 5-OP-RU or 6-FP tetramer (a kind gift from Dr. Jim McCluskey) to directly identify MAIT cells was performed at room temperature prior to the addition of surface antibodies. In some cases, cells were permeabilized with Transcription Factor staining buffer (BD biosciences) and stained intracellularly for the expression of Ki67, Tbet, PLZF (BD Biosciences), or Eomes (eBiosciences). For cellular function experiments, cells were stimulated, collected, washed, and incubated with live/dead green (Life Technologies), and anti-CD3, CD4, CD8, CD161, and Vα7.2 for 30 min at 4°C. Cells were permeabilized with Permeabilization/Fixation buffer (BD Biosciences) and incubated with anti-IFNγ, TNFα, IL-17, and GM-CSF (BD Biosciences) for 30 min at 4°C. Samples were run on a LSRII flow cytometer or LSRFortessa and data acquired with FACS Diva. Flow cytometry data were analyzed in FlowJo v10. A minimum of 50 events were required for analysis of phenotype or function of a given population. Stimulation responses are presented as background subtracted data.

### Plasma Protein Quantification

Frozen plasma was used to measure the expression of 17 cytokines and chemokines by multiplexed bead array, as described in Ref. (24). Briefly, the MILLIPLEX MAP Human Cytokine/Chemokine Magnetic Bead Panel (Millipore) was used to measure IFNα2, IL-1α, IL-1RA, IL-8, IFNγ-inducible protein (IP)-10, monocyte chemoattractant protein (MCP)-1, MCP-3, macrophage inflammatory protein (MIP)-1α, MIP-1β, sCD40L, and vascular endothelial growth factor, GM-CSF, IFNγ, IL-10, IL-1β, and TNFα. Plasma concentrations of C-reactive protein (CRP), soluble CD14 (sCD14), and LPS-binding protein (LBP) were quantified by commercial ELISA (R&D for CRP and sCD14, Hycult for LBP), as described in Ref. (24).

### Statistical Analysis

Univariate statistical analyses were performed in GraphPad Prism v6.0 (GraphPad). Groups were categorized based on ESRD and IGRA status. Two group comparisons were performed using the Mann–Whitney test. Four group comparisons were performed using the Kruskal–Wallis test, with differences between specific groups assessed by Dunn’s posttest. Categorical variables were compared using Fisher’s Exact Test. Correlations were performed using the Spearman test. Assessment of the contribution of ESRD and IGRA status as independent variables was performed by two-way ANOVA. Data are shown as box-and-whiskers plots, with the boxes indicating the median (line) and interquartile ranges, and the whiskers depicting the maximum and minimum values. All tests were two-sided, and *p* values < 0.05 were considered statistically significant. Multivariate analyses were performed in SAS v9.4 (SAS Institute). Multivariate linear regression analyses were performed using a backwards selection of the main effects model.

## Results

### Patient Demographics

The ESRD study participants were selected from a cohort established at the dialysis unit at the Health Sciences Centre in Winnipeg, MB, Canada, and non-ESRD controls were selected from a biobank of otherwise healthy IGRA positive and negative individuals recruited in the same city. This cohort has been previously described (23), as have the individuals selected for immunologic characterization (24) in this study (*n* = 10 in each group for ESRD−IGRA−, ESRD−IGRA+, ESRD+IGRA−, and ESRD+IGRA+). Participant demographics are summarized in Table 1.

**Table 1:** Characteristics of end-stage renal disease (ESRD) and non-ESRD participants, based on Interferon Gamma Release Assay (IGRA) status.

	ESRD−IGRA− (*n* = 10)	ESRD−IGRA+ (*n* = 10)	ESRD+IGRA− (*n* = 10)	ESRD+IGRA+ (*n* = 10)	*p-*Value
Median Age (IQR)					
Gender	53.5 (39.75, 59.5)	52 (39.25, 53.75)	59 (49.25, 69.25)	64.5 (48.75, 69.75)	>0.05
Female	6 (60%)	6 (60%)	7 (70%)	7 (70%)	>0.05
Male	4 (40%)	4 (40%)	3 (30%)	3 (30%)	
Ethnicity					
Canadian born	8 (80%)	5 (50%)	8 (80%)	10 (100%)	>0.05
Non-Canadian born	2 (20%)	5 (50%)	2 (20%)	0 (0%)	
BCG+, self-report	8 (*n* = 9, 88.9%)	5 (*n* = 8, 62.5%)	7 (70%)	10 (100%)	>0.05
Diabetes, self-report	4 (40%)	1 (10%)	5 (50%)	6 (*n* = 9, 66.7%)	>0.05
Cause of ESRD
Diabetes/diabetic nephropathy	–	–	4 (40%)	6 (60%)	–
IgA nephropathy	–	–	1 (10%)	0 (0%)	–
Cancer	–	–	0 (0%)	2 (20%)	–
Other	–	–	5 (50%)	2 (20%)	–

### ESRD Patients Exhibit Depletion of Peripheral Blood MAIT Cells

**Figure 1:**
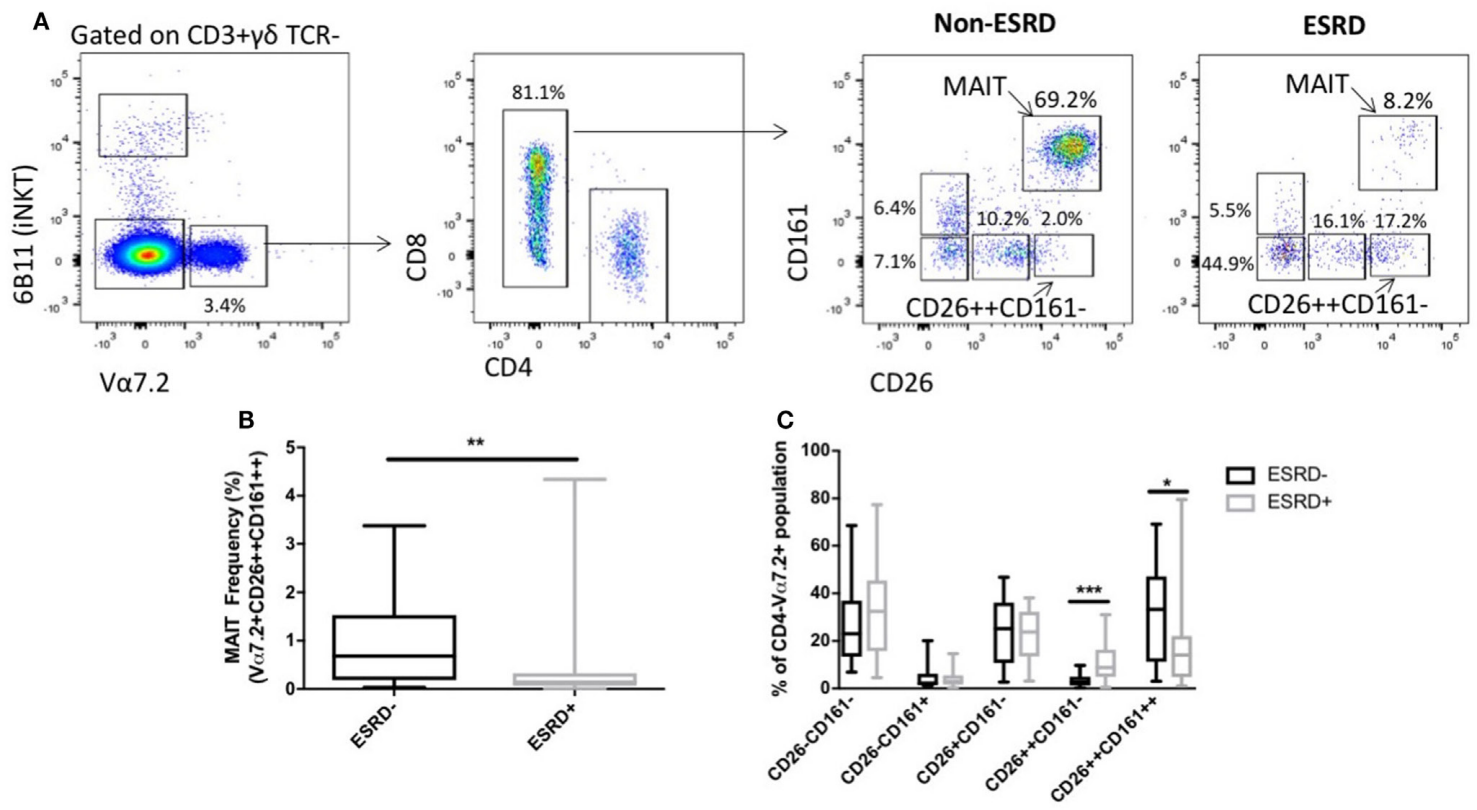
Identification of mucosal-associated invariant T (MAIT) cells using Vα7.2, CD161 and CD26 (*n* = 20 per group). **(A)** Representative staining of MAIT cell gating strategy. CD3+ γδ TCR−6B11− cells were gated for Vα7.2 expression. CD4−CD161++CD26++ cells were identified as MAIT cells. Other Vα7.2+CD4− populations were gated based on expression of CD161 and CD26. **(B)** Comparison of MAIT cell frequency (as a proportion of CD3+γδ TCR− cells) between non-end-stage renal disease (ESRD) and ESRD groups. **(C)** Comparison of the frequency of CD26 and CD161-expressing populations within the Vα7.2+CD4− gate. Comparisons performed by Mann–Whitney test, **p < 0.01, ***p < 0.001.

Mucosal-associated invariant T cells are most commonly defined based on the expression of the Vα7.2 TCR and high expression of CD161, and are almost exclusively CD8+ or CD4−CD8−. Several recent reports, however, have demonstrated that human MAIT cells also express uniformly high levels of CD26 (27), which can be useful in their identification, particularly in conditions such as TB infection where chronic stimulation might lead to CD161 downregulation (28). We, therefore, assessed MAIT cells using two approaches: characterization of MAIT cells expressing high levels of CD26 (CD3+γδ TCR−6B11−CD4−Vα7.2+CD161++CD26++), or characterization of conventional MAIT cells regardless of CD26 expression (CD4−Vα7.2+CD161++ cells). Assessment of CD26++ MAIT frequency among ESRD and non-ESRD participants as a proportion of CD3+γδ TCR− cells (Figure 1A) was significantly lower among ESRD participants compared to controls (*p* = 0.0097, Figure 1B). Analysis of conventional MAIT cells defined solely as CD4−Vα7.2+CD161++ showed the same significant reduction in frequency in ESRD patients, and correlated strongly with the frequencies determined when including CD26 in the gating strategy (Figures S1A,B in Supplementary Material). Although MAIT cells may decline with age (29), there was no correlation between age and MAIT cell frequency among either the ESRD or non-ESRD participants in this cohort (not shown).

One caveat to the identification of MAIT cells using surface markers such as CD161 and CD26 is the possibility that MAIT cell loss could be reflective either of true depletion, or simply the downregulation of CD161 on the cell surface (30). We, therefore, next assessed the frequency of CD26± and/or CD161± populations within the CD4−Vα7.2+ lymphocyte subset, and observed a significant accumulation of CD26++CD161− cells among ESRD participants (*p* = 0.0007, Figure 1C), which may represent MAIT cells that retained expression of Vα7.2 and CD26 but downregulated CD161.

**Figure 2:**
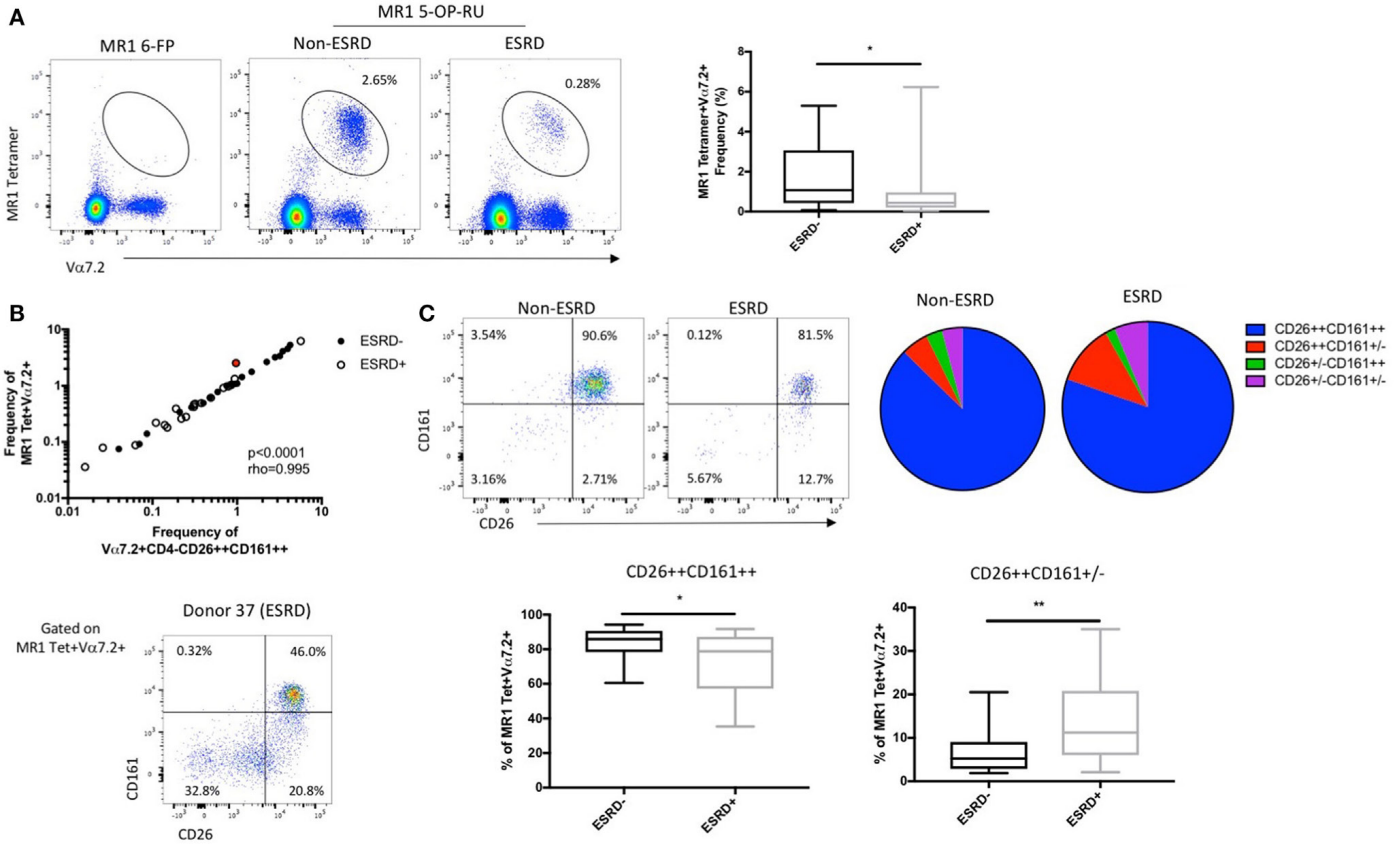
Comparison of mucosal-associated invariant T (MAIT) cell identification by surface marker expression and MR1 5-OP-RU tetramer binding (*n* = 20 per group). **(A)** Representative MR1 tetramer staining with the 6-FP (negative control) loaded tetramer or 5-OP-RU loaded tetramer, which binds to MR1-restricted MAIT cells, in a non-end stage renal disease (ESRD) and ESRD participant. The frequency of MR1 tetramer+Vα7.2+ T cells (as a proportion of CD3+Vδ2− T cells) is significantly reduced in ESRD participants compared to controls. **(B)** MAIT cell frequency (as a proportion of CD3+Vδ2− cells) as determined by MR1 tetramer binding strongly correlates with the frequency of Vα7.2+CD4−CD26++CD161++ cells (as a proportion of CD3+γδ TCR− cells) in both non-ESRD (closed circles) and ESRD participants (open circles). Donor 37, identified in red, exhibited a stronger discrepancy between MR1 tetramer frequency and surface marker frequency than other participants, due to a large proportion of MR1 tetramer+ cells with low CD26 and/or CD161 expression. **(C)** The expression of CD26 and CD161 on MR1 tetramer-staining cells was significantly altered between ESRD and control groups. ESRD participants exhibited a significantly lower proportion of CD26++CD161++ cells and an increase in CD26++CD161± cells compared to healthy controls. Comparisons performed by Mann–Whitney test, **p < 0.01, ***p < 0.001.

To determine whether CD161 downregulation was hindering MAIT cell identification in the ESRD cohort, we also performed additional staining using a human MR1 tetramer loaded with 5-OP-RU, which identifies all MR1-reactive T cells (31, 32), the vast majority of which express the Vα7.2 TCR (Figure 2A). An MR1 tetramer loaded with the 6-FP ligand (32, 33), which does not bind to MAIT cells, was used as a control for tetramer specificity (Figure 2A). Use of the MR1 5-OP-RU tetramer confirmed a significant depletion of peripheral blood MAIT cells in ESRD participants compared to controls (*p* = 0.027, Figure 2A) and showed a strong correlation with MAIT cell frequency identified by Vα7.2, CD26, and CD161 surface marker expression (*p* < 0.0001, Figure 2B).

While these results validate the use of surface markers to identify MAIT cells in ESRD patients, several individual ESRD participants did exhibit notable downregulation of CD26 and CD161 expression among the MR1 tetramer binding population (Figure 2C). Analysis of CD26 and CD161 expression on MR1 tetramer-positive cells among the whole cohort revealed a significant accumulation of CD26++CD161± cells among ESRD participants (*p* = 0.005, Figure 2C), consistent with the Vα7.2+CD26++CD161− population described in Figure 1. MAIT cells identified by the MR1 tetramer exhibited lower expression of CD26 than cells identified as CD4−Vα7.2+CD161++ (Figure S1C in Supplementary Material), confirming that the use of TCR and CD161 staining is slightly biased toward the identification of “canonical” MAIT cells. Overall, both the MR1 tetramer and Vα7.2/CD26/CD161 staining consistently demonstrate both the depletion of peripheral blood MAIT cells and the downregulation of CD161 on these cells in ESRD.

### MAIT Cells Are Activated but Retain NK Marker Expression in ESRD Patients

**Figure 3:**
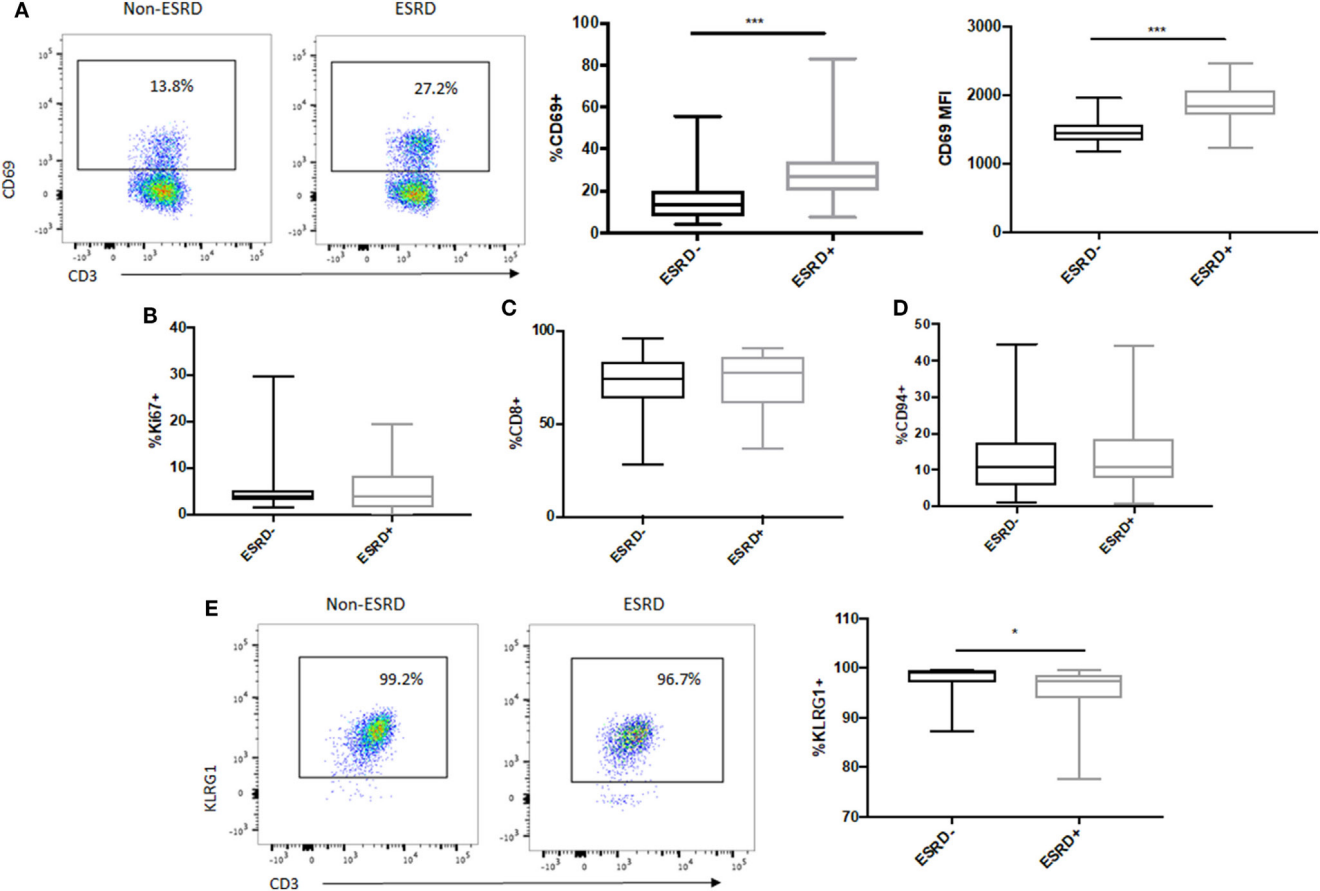
*Ex vivo* mucosal-associated invariant T (MAIT) activation and NK cell receptor expression. **(A)** Representative staining of the acute activation marker CD69 on control (*n* = 20) and end-stage renal disease (ESRD) (*n* = 19) MAIT cells (Vα7.2+CD26++CD161++). CD69 expression was significantly elevated among the ESRD group compared to healthy controls. **(B)** Comparable expression of Ki67 on MAIT cells among ESRD (*n* = 17) and non-ESRD (*n* = 16) controls. **(C)** The majority of MAIT cells in both groups were CD8+ (*n* = 20 control, *n* = 19 ESRD). **(D)** A small but consistent proportion of MAIT cells in both study groups expressed CD94 (*n* = 18 non-ESRD, *n* = 17 ESRD). **(E)** Representative staining of KLRG1 expression on MAIT cells in controls (*n* = 16) and ESRD participants (*n* = 17), which exhibited significantly lower KLRG1 expression. Comparisons performed by Mann–Whitney test, *p < 0.05, ***p < 0.001.

To assess the impact of ESRD on the remaining MAIT cell population, we first quantified MAIT activation, proliferation, and expression of surface markers previously associated with effector function. MAIT cell activation, as assessed by expression of the acute activation marker CD69, was significantly elevated among the ESRD group compared to controls, both as a proportion of CD69+ cells (*p* = 0.0002, Figure 3A) as well as the MFI of CD69 expression (*p* = 0.0002, Figure 3A). There was, however, no correlation between CD69 expression and CD26 or CD161 downregulation on MR1 tetramer-positive cells (not shown). This activation was also not associated with MAIT cell proliferation, based on low levels of Ki67 expression within the Vα7.2+CD26++CD161++ subset in all participants (*p* > 0.05, Figure 3B). In both groups, the majority of MAIT cells were CD8+ (Figure 3C), and a minority expressed CD94, part of the CD94/NKG2A heterodimer recently associated with enhanced MAIT cell cytokine production (22) (Figure 3D). Interestingly, while the vast majority of the MAIT population expressed the NK-associated cytotoxicity marker KLRG1 in both study groups, there was a statistically significant but minor drop in KLRG1 expression among ESRD patients (*p* = 0.027, Figure 3E).

### MAIT Cell Tissue Homing Receptor Expression Is Altered in ESRD

**Figure 4:**
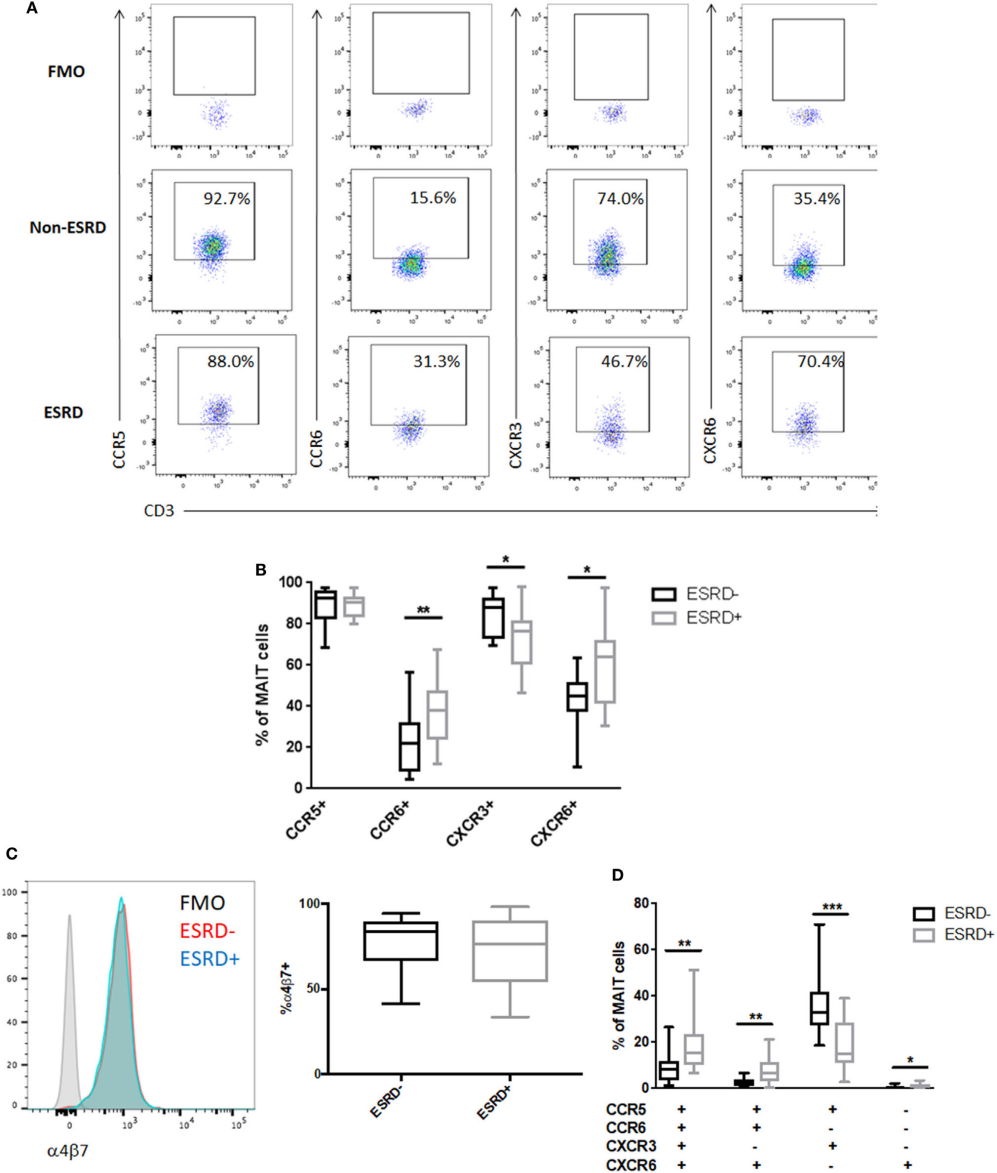
Mucosal-associated invariant T (MAIT) cell phenotype in end-stage renal disease (ESRD) (*n* = 16) and non-ESRD participants (*n* = 18). **(A)** Representative staining of MAIT cell (Vα7.2+CD26++CD161++) expression of CCR5, CCR6, CXCR3, and CXCR6 and fluorescence minus one (FMO) controls for each marker. **(B)** Comparison of the expression of CCR5, CCR6, CXCR3, and CXCR6 between non-ESRD and ESRD groups. **(C)** Maintenance of integrin α4β7 expression between ESRD and control groups. The histogram demonstrates representative α4β7 staining in each group compared to the FMO control. **(D)** Comparison of the frequency of MAIT cell populations determined by Boolean gating between non-ESRD and ESRD groups. Comparisons performed by Mann–Whitney test, *p < 0.05, **p < 0.01, ***p < 0.001.

Mucosal associated invariant T cells exhibit high tissue-homing capacity due to their expression of multiple chemokine receptors and integrins such as α4β7 (34). Analysis of bulk CD8+ T cell phenotype in non-human primate models of TB disease has demonstrated that lung-infiltrating T cells are highly enriched for the expression CCR5 and CXCR3 (35). These receptors, as well as CCR6 and CXCR6, are also involved in T cell recruitment to the kidney during renal disease (36). We, therefore, assessed the level of CCR5, CXCR3, CCR6, and CXCR6 expression on Vα7.2+CD26++CD161++ MAIT cells among study participants (Figure 4A). CCR5 expression was uniformly high among all samples, but ESRD-derived MAIT cells exhibited significantly reduced expression of CXCR3 (*p* = 0.016) and significantly elevated expression of CCR6 (*p* = 0.003) and CXCR6 (*p* = 0.013) compared to the non-ESRD group (Figures 4A,B). The significant differences between control and ESRD participant MAIT cells were observed regardless of whether CD26++ or total MAIT cells were considered (not shown). Similar to the relationship between MAIT frequency and age, there was no correlation between MAIT phenotype and age among the ESRD or non-ESRD groups (data not shown). In contrast to the altered expression of chemokine receptors on ESRD-derived MAIT cells, expression of the gut-homing integrin α4β7 was maintained among both healthy and ESRD participants (Figure 4C).

Boolean analysis of chemokine receptor co-expression identified four phenotypic subpopulations that were significantly altered in ESRD. The major CCR5+CCR6−CXCR3+CXCR6− MAIT cell phenotype observed in non-ESRD participants was significantly reduced among the ESRD group (*p* = 0.0004), which was instead accompanied by a significant increase in quadruple-positive MAIT cells (*p* = 0.0038), as well as CCR5+CCR6+CXCR3−CXCR6+ cells (*p* = 0.0024) (Figure 4D). Overall, the loss of CXCR3 expression on the bulk MAIT subset is primarily reflected by the loss of Th1-like CCR5+CXCR3+ MAIT cells. In contrast, the elevated proportion of CCR6+ MAIT cells may be reflective of an increase in inflammatory, Th17-like function.

### Shift From IFNγ to GM-CSF Production by PMA-Stimulated MAIT Cells in ESRD Patients

**Figure 5:**
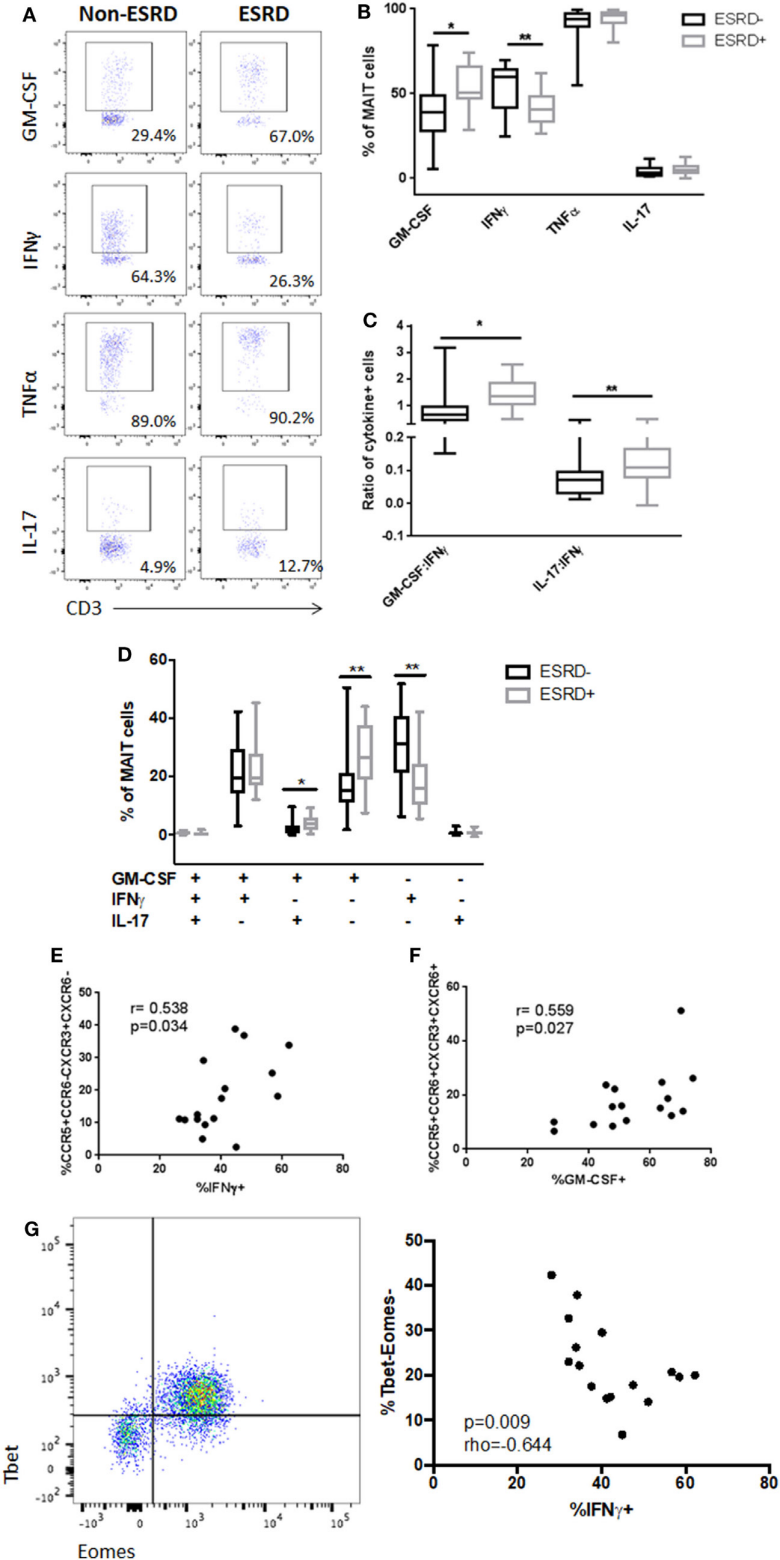
PMA-induced mucosal-associated invariant T (MAIT) cell (CD4−Vα7.2+CD161++) cytokine production [*n* = 19 end-stage renal disease (ESRD)−, *n* = 18 ESRD+]. **(A)** Representative staining of MAIT cell granulocyte macrophage-colony stimulating factor (GM-CSF), IFNγ, tumor necrosis factor α, and IL-17 production following 16 h stimulation with PMA/ionomycin. **(B)** Comparison of bulk cytokine production between ESRD and non-ESRD groups. **(C)** Comparison of the ratio of GM-CSF:IFNγ or IL-17:IFNγ production between ESRD and non-ESRD groups. **(D)** Comparison of polyfunctional cytokine responses determined by Boolean gating between ESRD and non-ESRD groups. **(E)** Correlation of bulk IFNγ production with surface marker expression among ESRD participants. **(F)** Correlation of bulk GM-CSF production with surface marker expression among ESRD participants. **(G)** Representative co-staining of Tbet and Eomes expression in the MAIT cell population and correlation between Tbet-Eomes- MAIT cell proportion and PMA-induced IFNγ production among ESRD participants (*n* = 16). Comparisons performed by Mann–Whitney test or Spearman correlation, *p < 0.05, **p < 0.01.

To determine whether alterations in surface marker expression were associated with functional changes in the MAIT subset, we stimulated PBMC cultures with PMA/ionomycin and assessed MAIT production of IFNγ, TNFα, IL-17, and the IL-17-associated cytokine GM-CSF (Figure 5A). As CD26 was not included in the cytokine flow panel, MAIT cells were defined as CD3+γδ TCR−CD4−Vα7.2+CD161++ cells. Following stimulation, production of both TNFα and IL-17 was similar, but there was a significant reduction in IFNγ production by ESRD-derived MAIT cells (*p* = 0.0036) that was accompanied by a significant increase in GM-CSF production (*p* = 0.018, Figure 5B). As a result, the ratio of Th17-like cytokines (IL-17 or GM-CSF) to Th1-like cytokines (IFNγ) was significantly elevated among ESRD participants (*p* = 0.020 for IL-17:IFNγ and 0.0010 for GM-CSF:IFNγ, Figure 5C). Boolean gating of IFNγ, GM-CSF, and IL-17 co-expression demonstrated that the primary perturbations of MAIT function in ESRD occurred in cells that expressed exclusively IFNγ or GM-CSF (TNFα was not included in the Boolean gating due to its near 100% expression level in all subjects). There was a significant loss of single-positive IFNγ+IL-17-GM-CSF− cells among ESRD participants (*p* = 0.0026), and an increase in single-positive IFNγ−IL-17-GM-CSF+ cells (*p* = 0.0032), as well as a minor population of IFNγ−IL-17+GM-CSF+ MAIT cells (*p* = 0.0168, Figure 5D).

Correlation of MAIT cytokine production and surface phenotype among ESRD participants confirmed that the loss of CXCR3 and the increased expression of CCR6 were associated with the observed changes in IFNγ and GM-CSF production. Bulk IFNγ production positively correlated with the frequency of CCR5+CCR6−CXCR3+CXCR6− cells (*p* = 0.034, Figure 5E), while bulk GM-CSF production correlated with the frequency of quadruple-positive cells (*p* = 0.027, Figure 5F), suggesting that MAIT cells expressing the highest levels of tissue homing markers were biased toward GM-CSF production and away from IFNγ production in ESRD patients.

Based on previous evidence that MAIT cell dysfunction is associated with the loss of key transcriptional regulators such as Tbet and Eomes (37), we assessed the transcription factor profile of MAIT cells in a subset of ESRD participants. While there was no relationship between PLZF expression (a master regulator of non-conventional T cells) (not shown) and MAIT function, the proportion of Tbet-Eomes- MAIT cells inversely correlated with PMA-induced IFNγ production (*p* = 0.009, Figure 5G).

Given the potential for MAIT cell function to decline with age, we also assessed the relationship between age and cytokine production in ESRD and non-ESRD controls. Interestingly, there was a significant inverse correlation between IFNγ production and age among non-ESRD participants (*p* = 0.0138), but no relationship between GM-CSF production and age among either ESRD or non-ESRD groups (Figure S2 in Supplementary Material).

### MAIT Responsiveness to Cytokines and Microbial Stimuli Is Altered in ESRD

**Figure 6:**
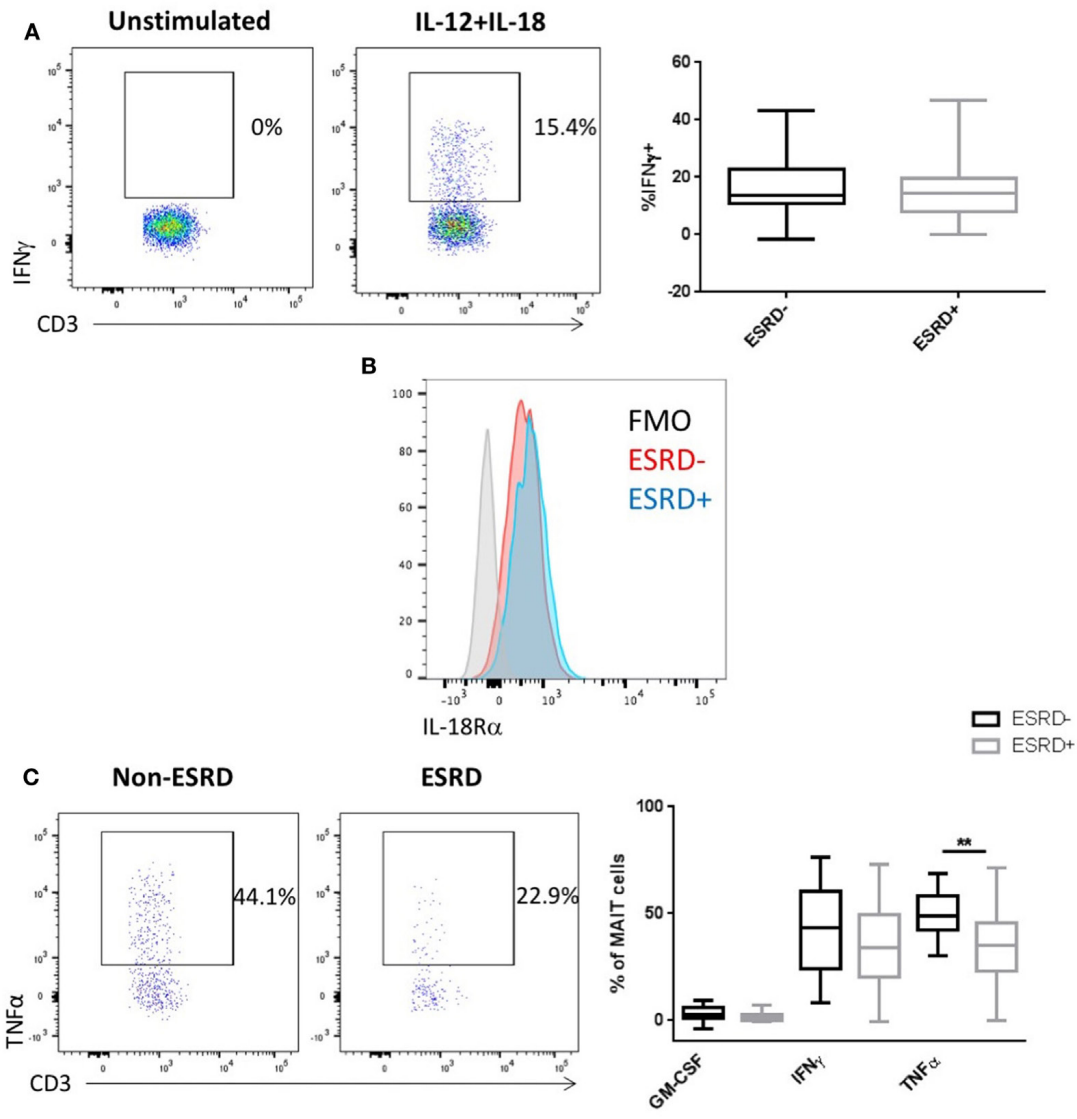
Mucosal-associated invariant T (MAIT) cell (CD4−Vα7.2+CD161++) responses to IL-12/IL-18 and *Escherichia coli* stimulation [*n* = 19 end-stage renal disease (ESRD)−, *n* = 18 ESRD+]. **(A)** Representative staining of IFNγ expression in an unstimulated control and IL-12/IL-18 stimulated sample, as well as a comparison of IFNγ production following 24 h IL-12/IL-18 stimulation between ESRD and non-ESRD groups. **(B)** Representative staining of IL-18Ra expression on MAIT cells in an ESRD and non-ESRD control sample as well as the fluorescent minus one (FMO) control. **(C)** Comparison of granulocyte macrophage-colony stimulating factor, IFNγ, and tumor necrosis factor α (TNFα) production following stimulation with *E. coli* and IL-15 between ESRD and non-ESRD groups. Representative staining shows TNFα production in response to stimulation in a non-ESRD and ESRD participant. Comparisons performed by Mann–Whitney test, **p < 0.01.

Mucosal associated invariant T cells, along with other CD161-expressing T cell subsets, can respond to both TCR-dependent antigenic stimulation or TCR-independent stimulation by cytokines such as IL-12 and IL-18 (38). We previously demonstrated that γδ T cells from ESRD patients exhibit hyporesponsiveness to IL-12/IL-18 stimulation (24) and, here, we assessed whether the MAIT cell subset would be similarly affected. Interestingly, ESRD had no impact on MAIT IFNγ production following cytokine stimulation (*p* > 0.05, Figure 6A), consistent with the maintenance of IL-18Rα expression *ex vivo* (Figure 6B). Thus, the impact of ESRD on MAIT function may depend on the pathway of cellular activation.

Stimulation of PBMC with fixed *E. coli* is a well-established assay to activate MAIT cells (39) in both an MR1-dependent (25) and cytokine-dependent manner (38), which can be further increased by the addition of exogenous IL-15 to culture (40). We, therefore, stimulated PBMC with either IL-15 alone or fixed *E. coli* plus IL-15. In contrast to PMA/Io, no IL-17 was induced by this stimulation, and only limited GM-CSF production was detectible. Surprisingly, *E. coli*-induced IFNγ production was comparable between ESRD and non-ESRD groups, but ESRD participants exhibited a significant reduction in TNFα production (*p* = 0.0013, Figure 6C). TNFα production in response to *E. coli* was substantially lower than that observed following PMA/Io, potentially explaining why no difference was observed in the context of the stronger mitogenic stimulation.

Given our previous observation that some MAIT cells exhibit CD161 downregulation in the ESRD cohort, we wondered whether those cells could retain their functionality. Although the MR1 tetramer was not available when the functional assays were performed, we gated on CD4−Vα7.2+CD161± cells and analyzed cytokine responses to IL-12/IL-18 or *E. coli* stimulation. Responses to IL-12/IL-18 stimulation were minimal and similar in both the control and ESRD groups (Figure S3A in Supplementary Material), but ESRD participants exhibited significantly lower responses to *E. coli* stimulation among the CD161± cells compared to controls (Figure S3B in Supplementary Material). The combination of elevated CD161± MAIT cells (evidenced by the MR1 tetramer) and decreased cytokine responses to stimulation among that population suggests that CD161± MAIT cells exhibit impaired function in response to microbial stimulation.

### Association Between Inflammation and MAIT Phenotype and Function in ESRD

**Figure 7:**
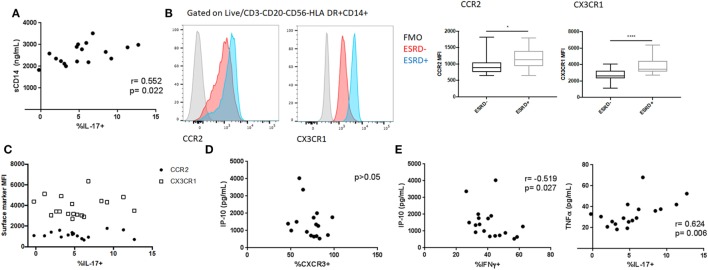
Relationship between inflammatory biomarkers and mucosal-associated invariant T (MAIT) cell (CD4−Vα7.2+CD161++) phenotype and function. **(A)** Correlation between PMA/ionomycin-induced MAIT IL-17 production and systemic sCD14 levels among end-stage renal disease (ESRD) participants. **(B)** Analysis of CCR2 (*n* = 16 ESRD−, *n* = 17 ESRD) and CX3CR1 (*n* = 20 per group) surface density on monocytes (defined as Live, CD3−CD20−CD56−HLA DR+CD14+ cells) among control and ESRD groups. **(C)** Correlation between monocyte phenotype and PMA/ionomycin-induced IL-17. **(D)** Correlation between MAIT cell CXCR3 expression and plasma IP-10 concentration among ESRD participants. **(E)** Correlation between PMA/ionomycin-induced IFNγ or IL-17 production and plasma cytokines among ESRD participants. Correlations performed by Spearman test. Comparisons performed by Mann–Whitney test, *p < 0.05, **p < 0.0001.

We previously reported elevated plasma levels of both sCD14 and CRP among the ESRD patients in this cohort (24). To determine the extent to which innate immune activation or inflammation was associated with alterations in MAIT phenotype or function, we conducted exploratory univariate correlations of these variables with overall MAIT frequency, the relative frequency of CXCR3+/CCR6+/CXCR6+/quadruple-positive MAITs, and PMA-induced cytokine responses (IFNγ, GM-CSF, IL-17) in ESRD patients. CRP did not correlate with any variables tested. sCD14 positively correlated with PMA-induced IL-17 production (*p* = 0.022, Figure 7A), suggesting that although MAIT IL-17 was not elevated among ESRD participants compared to controls, increased innate immune activation could drive elevated IL-17 secretion among some ESRD patients. As an alternate measure of innate immune inflammation, we quantified monocyte CCR2 and CX3CR1 expression among ESRD participants and controls (Figure 7B). Despite significantly elevated CCR2 and CX3CR1 surface density on ESRD patient monocytes, there was no correlation between monocyte phenotype and MAIT cell frequency or PMA-induced cytokine production (Figure 7C).

The ESRD cohort also exhibited elevated plasma levels of specific cytokines/chemokines, including IP-10, MCP-1, and TNFα (24). In particular, the elevated expression of IP-10, a CXCR3 ligand, could represent a mechanism underlying the altered expression of this chemokine receptor in ESRD. There was no correlation, however, between plasma IP-10 levels and MAIT CXCR3 expression among ESRD patients (Figure 7D), nor between plasma MCP-1 or TNFα and any other MAIT surface markers (not shown). In contrast, PMA-induced cytokine expression among ESRD patients was associated with plasma cytokine levels. MAIT IFNγ production inversely correlated with plasma IP-10 (*p* = 0.027), while IL-17 production positively correlated with plasma TNFα (*p* = 0.006) (Figure 7E). GM-CSF expression was not associated with any of the plasma cytokines.

Given the number of variables tested by univariate analysis, we also performed multivariate linear regression modeling to assess the relationship between inflammatory biomarkers and MAIT phenotype or function. A model containing CRP, PMA-induced IFNg and PMA-induced GM-CSF expression accounts for 49.9% of the variation in CXCR3 expression. It identified a significant inverse relationship of CXCR3 expression with plasma CRP (*p* = 0.044) and a trend toward an association with PMA-induced MAIT cell IFNγ expression (*p* = 0.072), confirming the univariate correlation shown in Figure 5E and implicating a relationship between elevated CRP levels and loss of surface CXCR3 (Table 2). As predicted by the bivariate analysis, age, gender, and diabetes status did not survive the backwards selection process (not shown). Modeling of variables associated with PMA-induced IFNγ identified inverse associations between cytokine production and plasma IP-10 (*p* = 0.025) and MCP-1 levels (*p* = 0.026; Table S2 in Supplementary Material), while modeling of PMA-induced IL-17 secretion demonstrated a positive association between cytokine production and plasma sCD14 (*p* = 0.037) and CRP (*p* = 0.0063) concentrations (Table S3 in Supplementary Material). Modeling of GM-CSF did not identify any significant relationships between inflammatory biomarkers and GM-CSF production. Overall, multivariate analysis suggests that ESRD participants with elevated inflammatory plasma profiles exhibit low surface CXCR3 expression and reduced IFNγ/elevated IL-17 expression upon PMA stimulation.

**Table 2:** Multivariate linear regression modeling of *ex vivo* mucosal-associated invariant T (MAIT) cell CXCR3 expression.

Dependent variable = MAIT CXCR3				
Independent variable	Bivariate correlation		Multivariate linear regression model	
			*R*^2^ = 0.4991	
	
	*r*	*p*	Parameter estimate	*p*
Plasma C-reactive protein	−0.31	0.286	−1.429	0.0435
PMA-stimulated IFNγ	0.45	0.106	0.621	0.0720
PMA-stimulated granulocyte macrophage-colony stimulating factor	0.033	0.919	0.406	0.162

### Minimal Impact of LTBI on ESRD-Associated MAIT Profile

We previously demonstrated that ESRD IGRA+ cohort participants exhibited substantial defects in γδ T cell function that were significantly different from the other study groups, including the control IGRA+ group, and instead, resembled the impaired γδ T cell function of active TB patients (24). Given the potential for MAIT cells to respond to Mtb infection, we wondered whether MAITs would similarly show functional differences in ESRD IGRA+ participants compared to the other study groups. Using two-way ANOVA to assess the separate impact or interaction of IGRA and ESRD status, we found no impact of IGRA positivity on the frequency of CCR5+CCR6+CXCR3−CXCR6+ MAIT cells (Figure S4A in Supplementary Material). Similarly, TNFα responses to *E. coli* stimulation were affected only by ESRD, but not IGRA, status (Figure S4B in Supplementary Material). In response to PMA stimulation, the frequency of IFNγ+ and GM-CSF+ cells was also unaffected by IGRA status (Figure S2C in Supplementary Material). Interestingly, there was a weak interaction between ESRD and IGRA status with respect to IL-17 production following PMA stimulation, due to low IL-17 production by non-ESRD IGRA+ participants compared to IGRA− controls, which was not observed among the ESRD IGRA+ group compared to ESRD IGRA− controls (Figure S4C in Supplementary Material, interaction *p* = 0.044).

## Discussion

Despite the multifaceted role of MAIT cells in responding to both bacterial and viral infection (9, 41), there are little data available to describe the impact of ESRD and inflammation on MAIT phenotype and function. We have demonstrated, for the first time, that MAIT cells exhibit phenotypic alterations in chemokine receptor expression and favor GM-CSF, rather than IFNγ, production following stimulation.

The reduced frequency of peripheral blood MAIT cells in ESRD patients is consistent with a previous report (13), but the comparison of MR1 tetramer-positive cells with Vα7.2+CD161++CD26++ T cells yielded interesting insights into the stability of MAIT cell surface marker expression in ESRD patients. It is clear that, at a cohort level, definition of MAIT cells as Vα7.2+CD161++CD26++ cells is a close approximation of the MR1-restricted T cell population. On an individual level, however, some ESRD patients exhibit substantial downregulation of CD161 and/or CD26 on MR1 tetramer-binding cells that could influence their identification by surface marker expression only. Although acute MAIT cell activation did not correlate with low MAIT frequency or loss of surface CD161 expression, it is possible that the study of chronic activation maker expression could yield more insight into the relationship between MAIT activation and loss of CD161 expression. It is also possible that the use of MR1 tetramers loaded with purified 5-OP-RU may not identify the entire MAIT cell compartment in individuals with chronic disease, given our increasing understanding of the variety of self- and foreign-antigens that may be presented by MR1 (42, 43); in future studies, tetramers loaded with heterogeneous bacterial lysate (44) or novel antigens may identify additional MAIT cells in these individuals.

The depletion of peripheral blood MAIT cells may be partially explained by the altered tissue homing receptor expression patterns among ESRD patients. While CXCR3 expression was lost on the MAIT population, expression of CCR6 and CXCR6 was increased among ESRD patients, which may result in differences in cell trafficking to tissues. The loss of CXCR3+CCR5+CCR6−CXCR6− MAIT cells (which predominated in healthy individuals) may be particularly detrimental to respiratory infections such as Mtb, as multiple studies have demonstrated a role for CCR5 and CXCR3 co-expression in trafficking of T cells to the lung during TB infection (16, 45). Recent mass cytometry analysis of tissue-resident CD8+ T cells (which would include MAIT cells) confirms the high expression of CCR5 and CXCR3 on lung-derived CD8+ cells (46). Although MAIT CXCR3 expression was lost overall, there was a significant elevation in the proportion of MAIT cells co-expressing all four chemokine receptors studied (CCR5, CXCR3, CCR6, CXCR6). These receptors, all of which are involved in cell trafficking to inflamed tissue, have been implicated in T cell recruitment to the kidney during glomerulonephritis (47) and cancer (36), and are highly expressed on MAIT cells infiltrating inflamed liver tissue (48). It is, therefore, possible that the alterations in MAIT phenotype among ESRD patients reflects their enhanced recruitment to inflamed tissues and disappearance from the circulation.

The loss of CXCR3 and increase in CCR6 expression were associated with alterations in MAIT cytokine production. Overall, the MAIT population in ESRD patients appeared to be biased away from a Th1-like (CXCR3+, IFNγ+) profile toward a Th17-like (CCR6+, GM-CSF+) profile. While we cannot exclude the possibility that alterations in functional profile are partially influenced by the use of CD161 and not CD26 to identify MAIT cells in the functional assays, the magnitude of the functional differences we observed was substantially greater than the proportion of MAIT cells that may have been excluded in a small number of participants. This shift in functional profile appears to be multi-factorial; the multivariate model including plasma sCD14, IP-10, MCP-1, and CRP levels accounted for 71% of the variation in PMA-induced IFNγ secretion, with the remainder of the variation at least partially attributable to the older age of the ESRD participants and the proportion of Tbet-Eomes- MAIT cells. Interestingly, loss of IFNγ-producing MAIT cells has recently been reported in type 1 diabetes (49). Diabetes is a prominent comorbidity and/or underlying cause of ESRD in this cohort, and future studies with greater power to compare diabetic and non-diabetic ESRD patients will be informative regarding the impact of diabetes on MAIT cell function prior to the progression of ESRD.

In contrast, the elevation of MAIT cell GM-CSF expression in this cohort was unrelated to either age, transcriptional profile, inflammatory biomarker levels, or monocyte phenotype, making it difficult to determine the mechanism underlying this shift in cytokine profile. Although data on MAIT cell GM-CSF production are limited in the current literature, excessive MAIT cell production of GM-CSF may further contribute to tissue inflammation during ESRD, given the increasing recognition of its role in other inflammatory processes (50–52); indeed, aberrant GM-CSF production by CD8+ T lymphocytes and gamma delta T cells has been reported in patients with inflammatory diseases such as spondyloarthritis (53). In recent years, T cell GM-CSF production has also been linked to RORγt expression and Th17-like cells (50), which play an important role in balancing the pro- and anti-inflammatory responses to Mtb in the lung. A nuanced understanding of the role that MAIT cell GM-CSF production might play in protection from ATB will require clarification of several points. First, it is unclear whether GM-CSF-producing MAIT cells are likely to traffic to the lungs, given the association between elevated GM-CSF production and increased CCR6/reduced CXCR3 expression on MAIT cells. If GM-CSF producing MAIT cells are present at the lungs; however, it is also unclear as to whether GM-CSF could mediate protection or pathology in the context of TB. *In vitro*, GM-CSF secretion by iNKT cells is associated with control of Mtb replication (20), and early production of GM-CSF by multiple T cell subsets contributes to immunity in mouse models (54). In individuals with pre-existing LTBI, however, increases in GM-CSF production in the lung will occur too late to contribute to control of infection and may instead contribute to immune pathology and inflammation. With the advent of technology to isolate and characterize the cytokine environment of individual sterile and necrotic granulomas (55), future studies in non-human primates will be positioned to quantify GM-CSF production at the site of Mtb replication.

Interestingly, there was no impact of LTBI status on MAIT function or phenotype in the context of ESRD. This stands in contrast to our observation that altered γδ T cell phenotype and function were associated with both ESRD and IGRA status in this cohort, suggesting that MAIT cells may be less sensitive to the presence of latent infection than other non-conventional T cell subsets. The ESRD-associated alterations in the MAIT population, however, may still impact host immunity to Mtb infection. Although IFNγ production may be maintained in ESRD patients through IL-12/IL-18 stimulation of MAIT cells, the lower levels of TNFα production in response to microbial stimulation point to defects in the response to bacterial infection. Anti-TNF therapy is a known risk factor for TB reactivation (56, 57), underscoring the importance of the TNF response in controlling Mtb. Together, the loss of CXCR3 expression, potential for MAIT cell homing to inflamed kidney tissues, and bias in cytokine production away from IFNγ/TNFα and toward GM-CSF production likely contribute to an immune environment that is more permissive to Mtb infection and reactivation.

This study describes, for the first time, significant alterations in MAIT frequency, phenotype, and function during ESRD that reflect systemic inflammation and altered responses to microbial stimulation. The loss of peripheral blood MAIT cells and a decrease in MAIT IFNγ secretion has now been reported in chronic HIV-1 infection (58), type 1 diabetes (53), and ESRD, all of which are associated with microbial translocation and/or loss of gut barrier integrity, as well as increased susceptibility to LTBI and TB reactivation. Future studies of MAIT functionality in earlier stages of CKD will provide better insight into the roles of inflammation and microbial translocation in MAIT cell impairment. Larger cohort studies will also provide further information on the impact of comorbidities commonly associated with ESRD, such as diabetes and obesity, on MAIT function. Additional study of MAIT GM-CSF production will provide critical insight into the induction of this cytokine during inflammation and its role in limiting or promoting Mtb infection and tissue damage.

## Ethics Statement

This study was carried out in accordance with the recommendations of the research ethics board at the University of Manitoba with written informed consent from all subjects. All subjects gave written informed consent in accordance with the Declaration of Helsinki. The protocol was approved by the research ethics board at the University of Manitoba.

## Author Contributions

JJ, JW, and KW performed the experiments. JJ analyzed the data and wrote the manuscript. CM and CL recruited study participants, and collected and processed clinical samples. JB organized patient recruitment and participated in study design. SK, TB, and SK conceived of and organized the study, and contributed to manuscript writing and editing. All authors contributed to manuscript revision, read and approved the submitted version.

## Conflict of Interest Statement

The authors declare that the research was conducted in the absence of any commercial or financial relationships that could be construed as a potential conflict of interest.

## Supplementary Material

Figure S1 Analysis of mucosal-associated invariant T (MAIT) cell frequency and phenotype using CD161 expression. **(A)** Statistical comparison of MAIT cell frequency between end-stage renal disease (ESRD) and control groups (*n* = 20 per group) by defining MAIT cells as CD4−Vα7.2+CD161++ cells. Statistics assessed by Mann–Whitney test. **(B)** Correlation between MAIT cell frequency defined as CD4−Vα7.2+CD161++ or CD4−Vα7.2+CD161++CD26++ cells among *n* = 40 cohort participants. Correlation performed by Spearman test. **(C)** Comparison of CD26++ expression on MAIT cells when identified by Vα7.2 and CD161 expression versus MR1 tetramer staining. *N* = 20 each in the control and ESRD cohorts. Statistics assessed by Wilcoxon test. **p* < 0.05, ***p* < 0.01, ****p* < 0.001.

Figure S2 Correlation between mucosal-associated invariant T PMA-induced cytokine production and age among end-stage renal disease (ESRD) and non-ESRD participants.

Figure S3 Cytokine production by CD4−Vα7.2+CD161± cells. **(A)** Comparison of IFNγ secretion following IL-12/IL-18 stimulation in the CD4−Vα7.2+CD161± population between end-stage renal disease (ESRD)− and ESRD+ groups (*n* = 20 ESRD− and 19 ESRD+). **(B)** Comparison of IFNγ, tumor necrosis factor α, and granulocyte macrophage-colony stimulating factor production by CD4−Vα7.2+CD161± cells following stimulation with *Escherichia coli* and IL-15 (*n* = 20 per group). Statistics assessed by Mann–Whitney test. **p* < 0.05, ***p* < 0.01.

Figure S4 Analysis of mucosal-associated invariant T (MAIT) phenotype and function based on end-stage renal disease (ESRD) and interferon gamma release assay (IGRA) status. Two-way analysis of separate impact of ESRD status and IGRA status on **(A)** the frequency of quadruple positive (CCR5+CCR6+CXCR3+CXCR6+) MAIT cells, **(B)** the frequency of tumor necrosis factor α+ MAIT cells in response to *Escherichia coli* and IL-15 stimulation, and **(C)** the frequency of granulocyte macrophage-colony stimulating factor, IFNγ or IL-17+ MAIT cells in response to PMA/Ionomycin stimulation. Comparisons performed by two-way ANOVA with Sidak’s multiple comparisons posttest.
