# Supplementary figures and images for: Mucosal-Associated Invariant T Cells Are Depleted and Exhibit Altered Chemokine Receptor Expression and Elevated Granulocyte Macrophage-Colony Stimulating Factor Production During End-Stage Renal Disease
Source: Front Immunol. 2018 May 17;9:1076. doi: 10.3389/fimmu.2018.01076 (PMC5967229; doi:10.3389/fimmu.2018.01076)

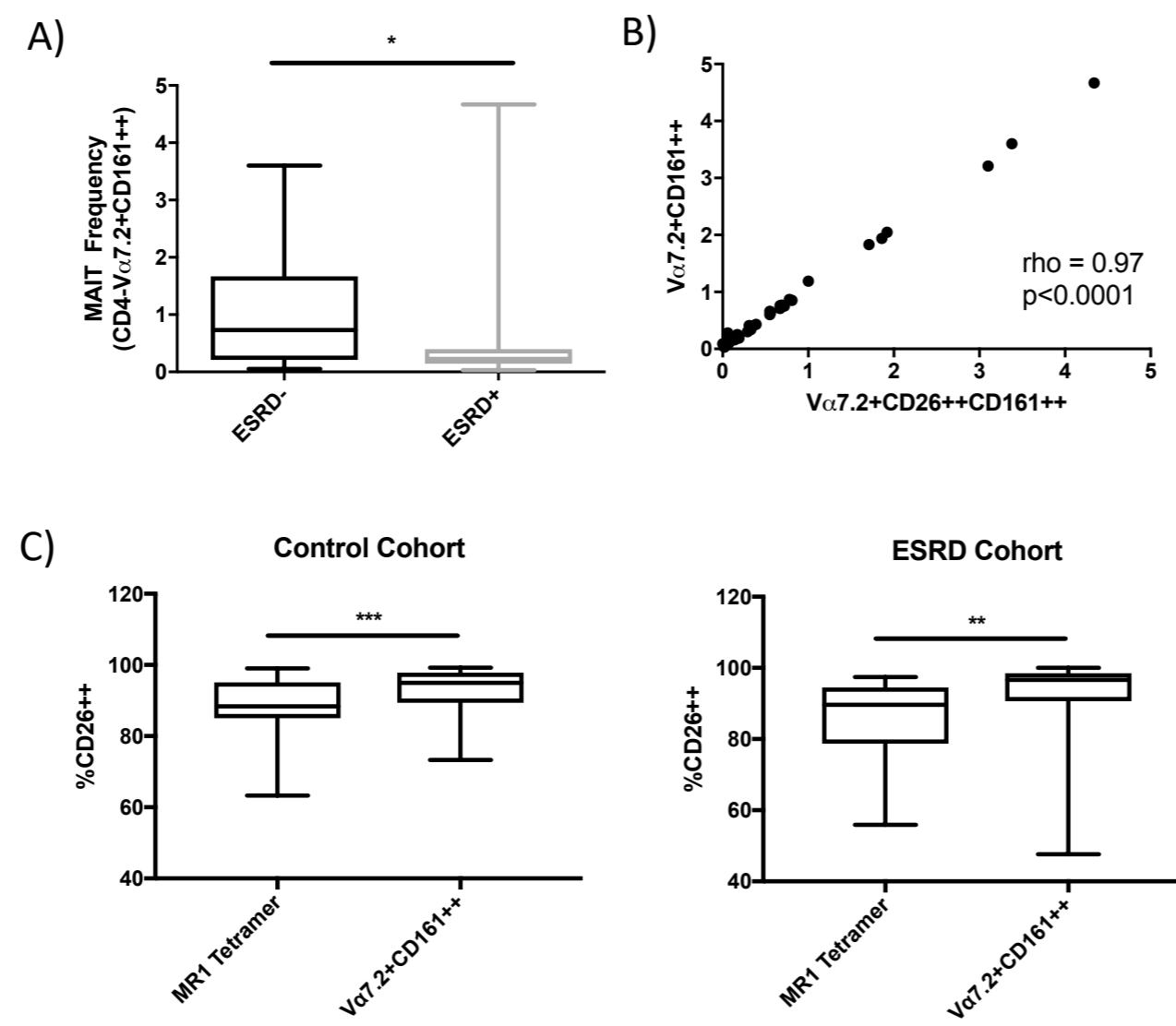

Supplementary Figure 1

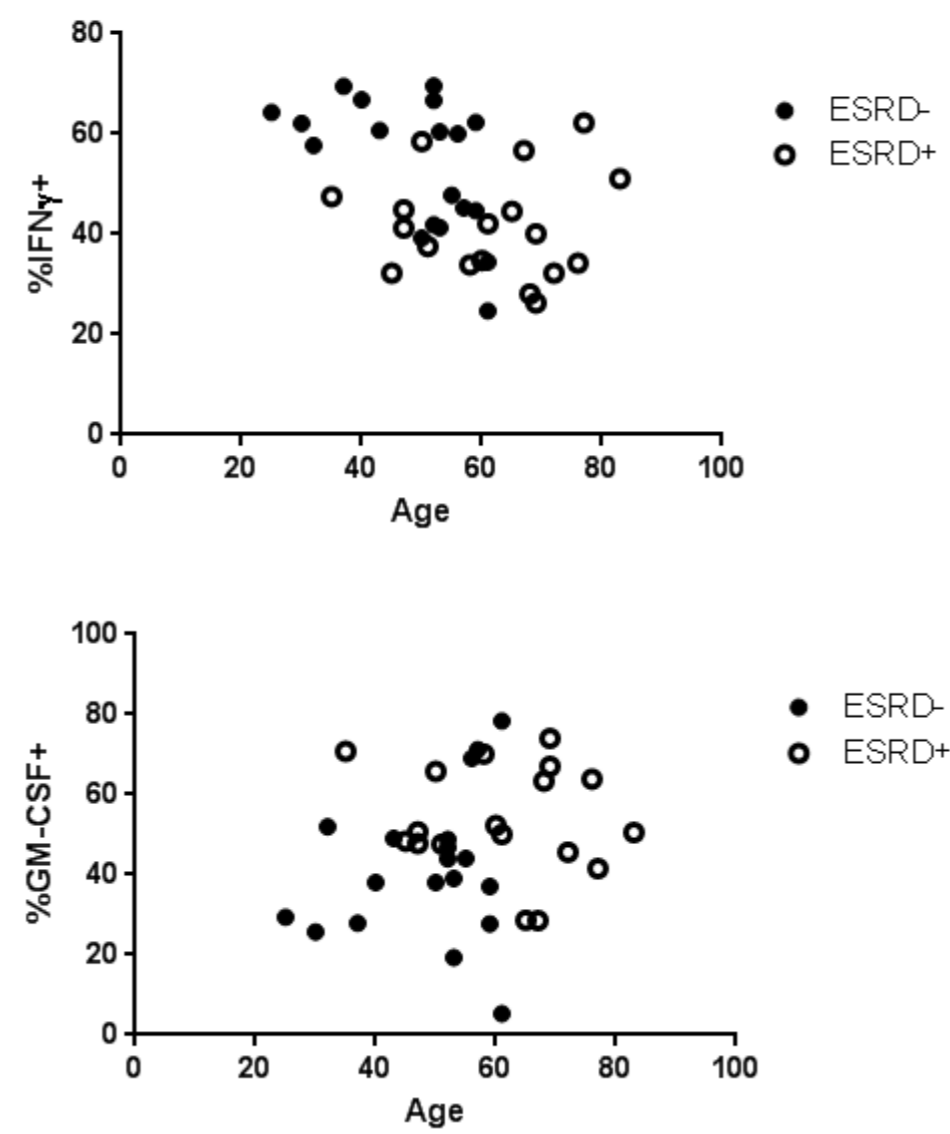

Supplementary Figure 2

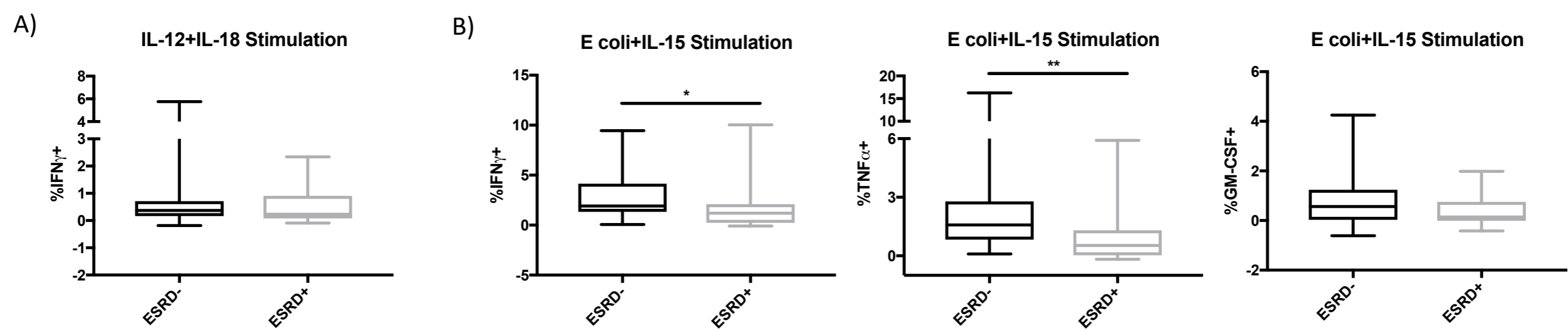

Supplementary Figure 3

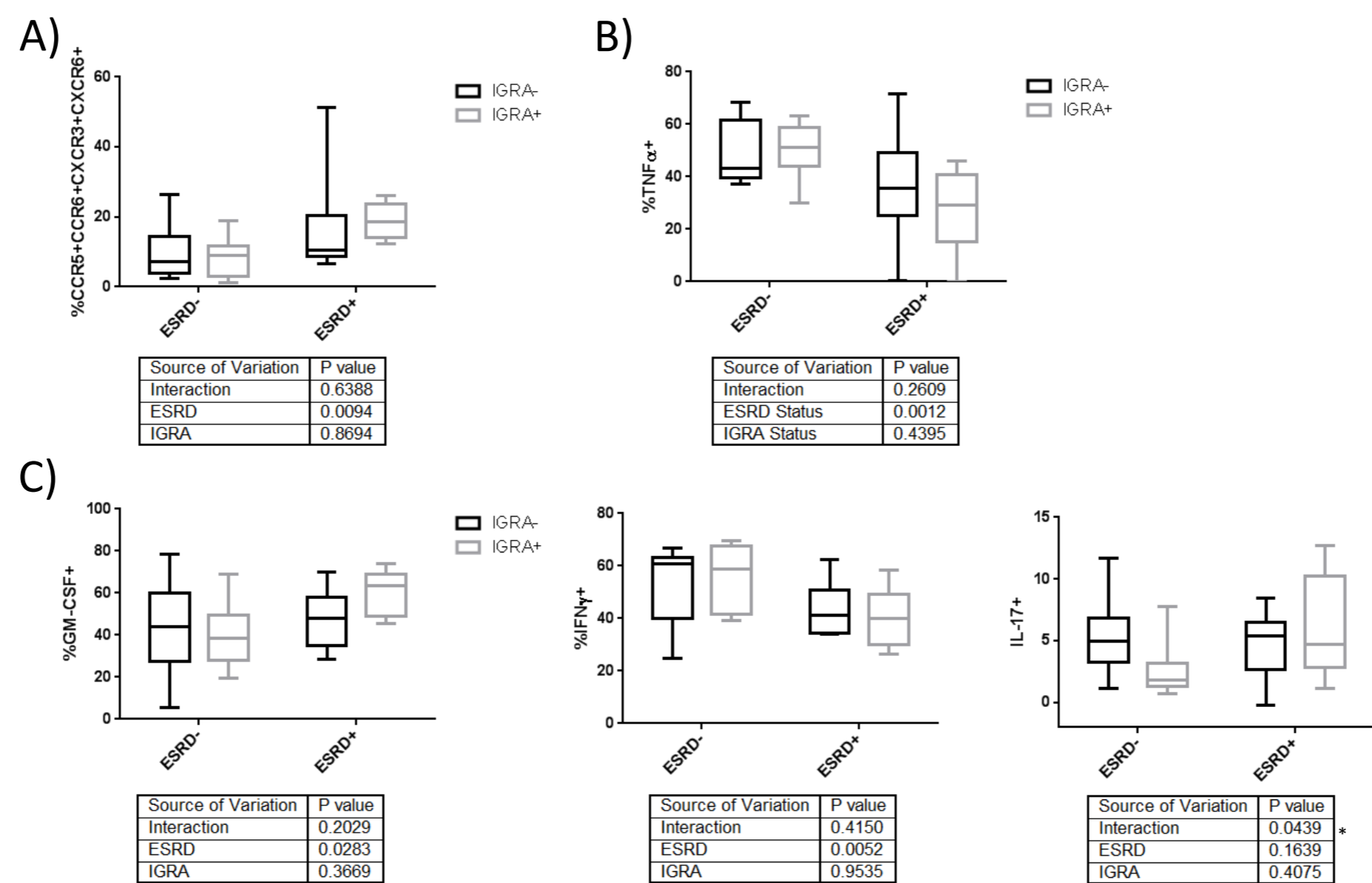

Supplementary Figure 4

Supplement: Figure S1 — Analysis of mucosal-associated invariant T (MAIT) cell frequency and phenotype using CD161 expression. (A) Statistical comparison of MAIT cell frequency between end-stage renal disease (ESRD) and control groups (n = 20 per group) by defining MAIT cells as CD4−Vα7.2+CD161++ cells. Statistics assessed by Mann–Whitney test. (B) Correlation between MAIT cell frequency defined as CD4−Vα7.2+CD161++ or CD4−Vα7.2+CD161++CD26++ cells among n = 40 cohort participants. Correlation performed by Spearman test. (C) Comparison of CD26++ expression on MAIT cells when identified by Vα7.2 and CD161 expression versus MR1 tetramer staining. N = 20 each in the control and ESRD cohorts. Statistics assessed by Wilcoxon test. *p < 0.05, **p < 0.01, ***p < 0.001. [file image_1.PDF]
