# Supplementary material for: Mucosal-Associated Invariant T Cells Are Depleted and Exhibit Altered Chemokine Receptor Expression and Elevated Granulocyte Macrophage-Colony Stimulating Factor Production During End-Stage Renal Disease
Source: Front Immunol. 2018 May 17;9:1076. doi: 10.3389/fimmu.2018.01076 (PMC5967229; doi:10.3389/fimmu.2018.01076)
Supplement: Supplementary file 2 [file table_1.PDF]

Supplementary Table 1. Antibodies used in flow cytometry staining

Marker	Fluorochrome(s)	Clone	Isotype
α4β7	BV421	Act-1	Ms IgG1
CCR5	Alexa700	J418F1	Rat IgG2b
CCR6	BV786	140706	Ms IgG1
CD161	BV421, APC-Fire 750	HP-3G10	Ms IgG1
CD26	APC, FITC	BA5b	Ms IgG2a
CD3	Alexa700, APC-H7	SK7	Ms IgG1
CD4	BV605	RPA-T4	Ms IgG1
CD69	APC-Fire 750	FN50	Ms IgG1
CD8	BV650	RPA-T8	Ms IgG1
CD94	BUV395	HP-3D9	Ms IgG1
CXCR3	PE-Dazzle 594	G025H7	Ms IgG1
CXCR6	APC	K041E5	Ms IgG2a
IL-18Ra	APC	H44	Ms IgG1
KLRG1	PeCy7	13F12F2	Ms IgG2a
Vα7.2	BV510, PerCP-Cy5.5	3C10	Ms IgG1
GM-CSF	PE-CF594	BVD2-21C11	Rat IgG2a
IFNγ	BV510	B27	Ms IgG1
IL-17A	BV786	N49-653	Ms IgG1
TNFα	Alexa700	MAb11	Ms IgG1
Eomes	PE eFluor610	WD1928	Ms IgG1
Ki67	BUV395	B56	Ms IgG1
Tbet	BV421	4B10	Ms IgG1

Ms, mouse

Supplementary Table 2. Multivariate linear regression modeling of ESRD MAIT cell PMA-induced IFNγ expression

Dependent variable = PMA-stimulated IFNγ

Independent variable	Multivariate linear regression model **R^2=0.7119**	
	Parameter estimate	p
Plasma sCD14	0.0124	0.1057
Plasma IP-10	-0.0098	**0.0249**
Plasma MCP-1	-0.0160	**0.0255**
Plasma CRP	0.8326	0.1169

Supplementary Table 3. Multivariate linear regression modeling of ESRD MAIT cell PMA-induced IL-17 expression

Dependent variable = PMA-stimulated IL-17

Independent variable	Multivariate linear regression model **R^2=0.6766**	
	Parameter estimate	p
Plasma sCD14	0.0042	**0.0372**
Plasma MCP-1	-0.0015	0.3576
Plasma CRP	0.4451	**0.0063**
